# Efficacy of pericapsular nerve group block vs. fascia iliaca compartment block for Hip surgeries: A systematic review and meta-analysis

**DOI:** 10.3389/fsurg.2023.1054403

**Published:** 2023-02-10

**Authors:** Haifeng Ying, Lingyang Chen, Danyang Yin, Yongqing Ye, Jian Chen

**Affiliations:** Department of Anesthesiology, Taizhou Hospital of Zhejiang Province Affiliated to Wenzhou Medical University, Linhai, China

**Keywords:** hip surgery, analgesia, nerve block, pain, anesthesia

## Abstract

**Objective:**

The review aimed to compare outcomes of pericapsular nerve group block (PENG) vs. fascia iliaca compartment block (FICB) for patients undergoing hip surgeries.

**Methods:**

Randomized controlled trials (RCTs) published in the databases of PubMed, CENTRAL, Embase, and Web of Science comparing PENG vs. FICB for pain control after hip surgeries were included in the review.

**Results:**

Six RCTs were included. 133 patients received PENG block and were compared with 125 patients receiving FICB. Our analysis showed no difference in 6 h (MD: −0.19 95% CI: −1.18, 0.79 *I*^2 ^= 97% *p* = 0.70), 12 h (MD: 0.04 95% CI: −0.44, 0.52 *I*^2 ^= 72% *p* = 0.88) and 24 h (MD: 0.09 95% CI: −1.03, 1.21 *I*^2 ^= 97% *p* = 0.87) pain scores between PENG and FICB groups. Pooled analysis showed that mean opioid consumption in morphine equivalents was significantly less with PENG as compared to FICB (MD: −8.63 95% CI: −14.45, −2.82 *I*^2 ^= 84% *p* = 0.004). Meta-analysis of three RCTs showed no variation in the risk of postoperative nausea and vomiting in the two groups. The quality of evidence on GRADE was mostly moderate.

**Conclusion:**

Moderate quality of evidence suggests that PENG may result in better analgesia than FICB in patients undergoing hip surgeries. Data on motor-sparing ability and complications are scarce to draw conclusions. Further large-scale and high-quality RCTs should be conducted to supplement current findings.

**Systematic Review Registration:**

https://www.crd.york.ac.uk/prospero/, identifier CRD42022350342.

## Introduction

Hip surgeries are commonly performed for degenerative diseases and traumatic indications worldwide (1). Irrespective of the type of surgical procedure, hip surgeries cause significant postoperative pain which can result in further complications and patient dissatisfaction. Indeed, the need for optimal analgesia cannot be underestimated for hip surgeries wherein the patient clientele is mostly elderly and the joint is primarily involved in patient mobility. Opioids are commonly used for postoperative analgesia but considering the associated complications like nausea, vomiting, delirium, constipation, and respiratory depression (2), regional anesthetic techniques are increasingly being used to provide better pain control (3).

The femoral nerve block and the fascia iliaca compartment block (FICB) are commonly used analgesic techniques in hip surgeries. Both blocks have equivalent analgesic efficacy with the femoral block being administered at the femoral crease just lateral to the femoral vessels (4). On the other hand, the FICB is a block wherein high volumes of local anesthetic is introduced below the fascia iliaca to achieve diffusion of the solution along the psoas muscle targeting the femoral nerve, lateral femoral cutaneous nerve, and the obturator nerve. However, a disadvantage of the block is the associated motor weakness of the surgical limb which can delay recovery and patient discharge (5). Recently, Girón-Arango et al. (6) have described a novel anesthetic modality named the pericapsular nerve group (PENG) block which anesthetizes the femoral nerve, obturator nerve, and the accessory obturator nerve while sparing the motor components. Studies have shown that the PENG block is efficient in providing postoperative analgesia in patients undergoing hip surgeries with preservation of quadriceps muscle strength (7, 8). However, the question remains what is the efficacy of PENG block vis-à-vis FICB which is commonly used in hip surgery patients? In this context, a number of trials have been published in the past few years comparing the two techniques for hip surgery patients (9–11). However, there has been no systematic review which has been conducted till date. Owing to this deficiency in literature, we present the first meta-analysis to compare the analgesic efficacy of PENG block vs. FICB for hip surgery patients.

## Material and methods

The systematic review was registered online on PROSPERO (CRD42022350342). The protocol was registered as a comparison of PENG vs. the femoral group of nerve blocks (femoral nerve block and FICB) for hip surgeries. However, on a literature search, it was found that most trials compared PENG vs. FICB, and only one trial compared PENG with the femoral nerve block. Hence, the current review was restricted to a comparison of PENG vs. FICB only.

### Search and eligibility

The PRISMA guidelines were followed for reporting the review (12). A comprehensive and systematic literature search strategy was devised for the databases of PubMed, CENTRAL, Embase, and Web of Science for trials related to the study. The search was last conducted on 5th August 2022 with no restriction on the date of publication or the article language. We used the following search terms, namely, “pericapsular nerve group block”, “fascia iliaca compartment block”, “PENG”, “hip”, and “surgery”. The search strings which were the same for all databases are shown in Supplementary Table S1. The studies which were searched were organized and duplicates were removed. After initial title and abstract screening by two reviewers of all deduplicated literature, only those relevant to the review were downloaded and further screened as per the eligibility criteria. All studies completing the criteria were eligible.

The inclusion criteria based on PICOS was:

- Population: Any type of hip surgery patients
- Intervention: PENG block
- Comparison: FICB
- Outcomes: Any one of the following- pain scores, total analgesic consumption, or time to first analgesic request after surgery
- Study type: Randomized controlled trials (RCTs)

We excluded studies analysing the efficacy of PENG for patient positioning after hip fractures. We further did not include observational studies, review articles, and editorials.

The literature search and screening were carried out separately by the two reviewers. All differences were resolved in consultation with a third reviewer.

### Data extraction

Names of study authors, publication year, trial region, type of hip surgery, sample size, gender details, the protocol of baseline anesthesia, PENG and FICB, use of rescue analgesics, and outcome data were extracted from the studies. Data on pain scores, total analgesic consumption, time to first analgesic request, and complications were analyzed.

The Cochrane Collaboration risk of bias-2 tool was used to judge the quality of RCTs (13). All studies were given ratings as low risk, high risk, or some concerns for each of the following domain of the risk of bias-2 tool, namely, randomization process, deviation from intended intervention, missing outcome data, measurement of outcomes, selection of reported result, and overall risk of bias. Grading of Recommendations Assessment, Development, and Evaluation (GRADE) tool based on the GRADEpro GDT software [GRADEpro Guideline Development Tool. McMaster University, 2020 (developed by Evidence Prime, Inc.)] was utilized to assess the certainty of the evidence.

### Statistical analysis

Continuous data were sourced to generate mean difference (MD) with 95% confidence intervals (CI). When data was in graphical format, Engauge Digitizer Version 12.1 was used to generate data. In case data was reported as median and range, it was converted using the formula published by Wan et al. (14). Separate analyses for pain scores were conducted based on the most commonly reported follow-up intervals (6 h, 12 h, and 24 h). Total analgesic consumption was converted into oral morphine equivalents using the opioid conversion table from the Faculty of Pain Medicine of the Australian and New Zealand College of Anesthetists (15). Complication data were compared to obtain odds ratios (OR).

A sensitivity analysis was done wherein we removed one study at a time to further check the results. Subgroup analysis was conducted based on type of FICB (suprainguinal or infrainguinal) if sufficient studies were available. The *I*^2^ statistic was used to explore between-study heterogeneity. Since the number of studies were less than 10, we did not use funnel plots for publication bias. “Review Manager” [RevMan, version 5.3; Nordic Cochrane Centre (Cochrane Collaboration), Copenhagen, Denmark; 2014] was chosen for the meta-analysis.

## Results

**Figure 1:**
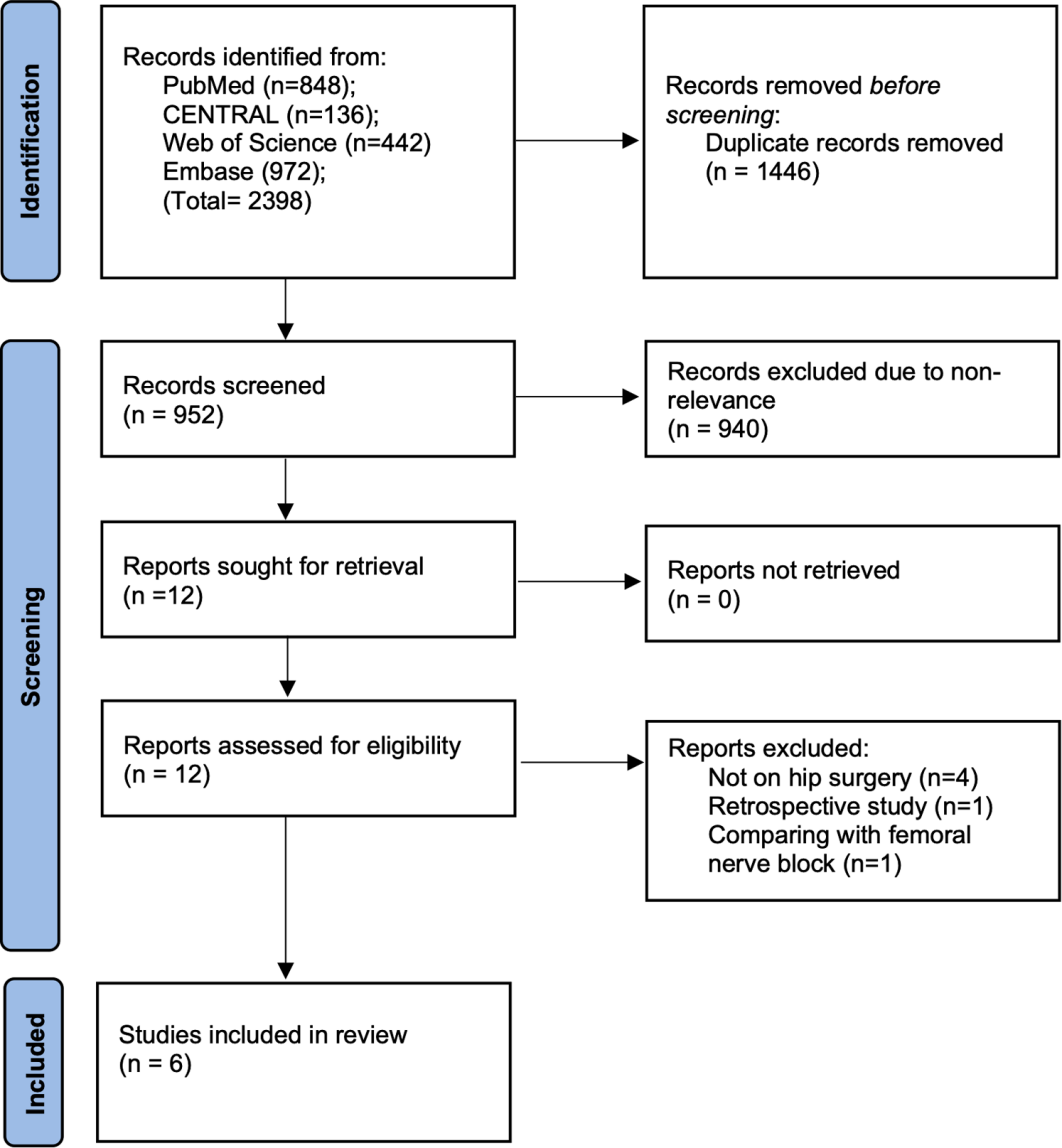
Study flow chart.

Search of all databases led to 2,398 articles of which 1,446 were excluded due to duplicity. On examining 952 studies, 12 records were chosen for complete text analysis and downloaded. Of these, six were excluded due to reasons listed in Figure 1, and the remaining six were included in this study (Figure 1) (9–11, 16–18).

The included studies were all RCTs conducted in India, China, Iran, Korea, and Chile (Table 1). All trials were published in 2021–22. Except for one study which used general anesthesia (10), all trials were conducted with baseline spinal anesthesia. Two studies (9, 18) administered the blocks after surgery while the remaining administered them before surgery. Both blocks were carried out under ultrasonography (USG) guidance in all trials. Two studies used suprainguinal FICB while the remaining used infrainguinal blocks. Either ropivacaine or levobupivacaine was used for the blocks. 133 patients received PENG block in the six trials and were compared with 125 patients receiving FICB. The various hip surgeries carried out were either dynamic screw fixation, nailing, hemi or total arthroplasty. All studies except one (17) used opioids for rescue analgesia. The study of Natarajan et al. (17) used paracetamol for rescue analgesia but did not report total analgesic consumption and hence could not be included in the meta-analysis. The trial (17) also failed to report standard deviation values of pain scores. Correspondence to authors for missing data went unanswered.

**Table 1:** Details of included studies.

Study	Location	Baseline anesthesia	Timing of block	PENG group	FICB group	Sample size	Male Gender (%)	Surgery types	Rescue analgesic		
						PENG	FICB	PENG	FICB		
Senthil 2022 (18)	India	Spinal with 3–3.5 ml of 0.5% bupivacaine	After surgery	USG guided block with 30 ml 0.25% levobupivacaine and 4 mg dexamethasone	USG guided infrainguinal block with 30 ml 0.25% levobupivacaine and 4 mg dexamethasone	20	20	50	60	DHS fixation, proximal femur nailing	PCA of fentanyl infusion bolus of 20 μg, maximal hourly dose limit of 100 μg, and lockout interval of 10 min
Natarajan 2022 (17)	India	Spinal	Before surgery	USG guided block with 20 ml 0.5% ropivacaine	USG guided infrainguinal block with 20 ml 0.5% ropivacaine	12	12	NR	NR	DHS fixation, hemiarthroplasty	Injection paracetamol 1 g IV
Mosaffa 2022 (16)	Iran	Spinal	Before surgery	USG guided block with 3 ml/kg 0.5% ropivacaine	USG guided infrainguinal block with 3 ml/kg 0.5% ropivacaine	30	22	73.3	72.7	DHS fixation, gamma nail, screw fixation	PCA of morphine, 1 mg with every push, 15 min lockout interval
Hua 2022 (11)	China	Spinal with 2–2.5 ml of 0.5% bupivacaine	Before surgery	USG guided block with 20 ml 0.4% ropivacaine	USG guided infrainguinal block with 30 ml 0.4% ropivacaine	24	24	58.3	54.1	Hemi or total hip arthroplasty	PCA of sufentanil, 1 μg/h background infusion, 2 μg with every push, 15 min lockout interval
Choi 2022 (10)	Korea	General	Before surgery	USG guided block with 20 ml 0.2% ropivacaine with epinephrine 1:200,000	USG guided suprainguinal block with 30 ml 0.2% ropivacaine with epinephrine 1:200,000	27	27	51.9	59.3	Total hip arthroplasty	PCA of fentanyl 7 μg/kg in 100 ml saline, 2 ml/h background infusion, 0.5 ml with every push, 15 min lockout interval
Aliste 2021 (9)	Chile	Spinal with 2 ml of 0.5% bupivacaine and 20 μg of fentanyl	After surgery	USG guided block with 20 ml 0.5% levobupivacaine with epinephrine 5 μg/ml	USG guided suprainguinal block with 40 ml 0.25% levobupivacaine with epinephrine 5 μg/ml	20	20	35	35	Total hip arthroplasty	PCA of morphine, 1 mg with every push, 8 min lockout interval

DHS, dynamic hip screw; FICB, fascia iliaca compartment block; IV, intravenous; PCA, patient controlled analgesia; PENG, pericapsular nerve group block; USG, ultrasonography.

### Meta-analysis

**Figure 2:**
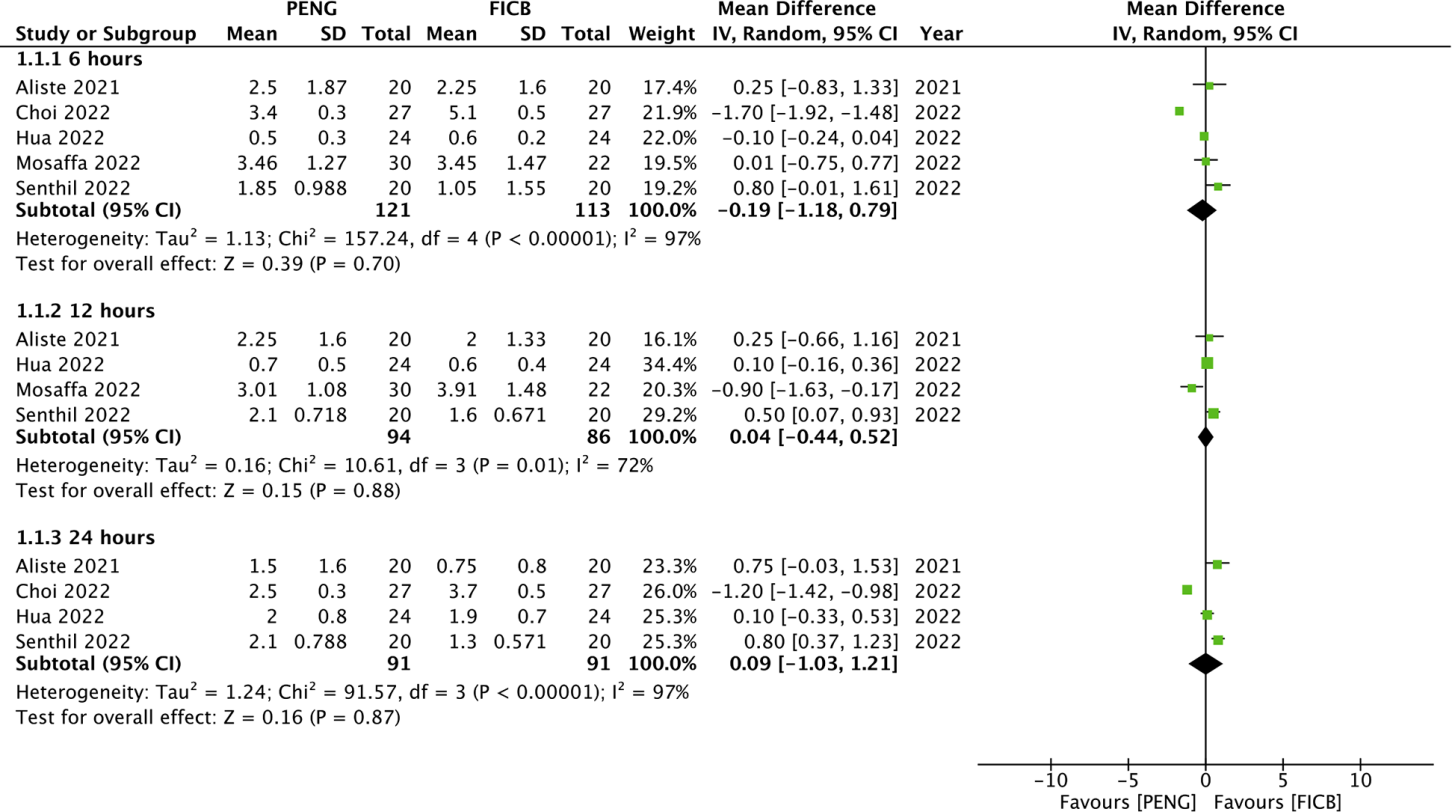
Meta-analysis of pain scores at 6 h, 12 h and 24 h between PENG and FICB groups.

Pain measured on a 10-point scale was available from five trials. Our analysis showed no difference in 6 h (MD: −0.19 95% CI: −1.18, 0.79 *I*^2 ^= 97% *p* = 0.70), 12 h (MD: 0.04 95% CI: −0.44, 0.52 *I*^2 ^= 72% *p* = 0.88) and 24 h (MD: 0.09 95% CI: −1.03, 1.21 *I*^2 ^= 97% *p* = 0.87) pain scores between PENG and FICB groups (Figure 2). All results were unchanged on sensitivity analysis.

**Figure 3:**
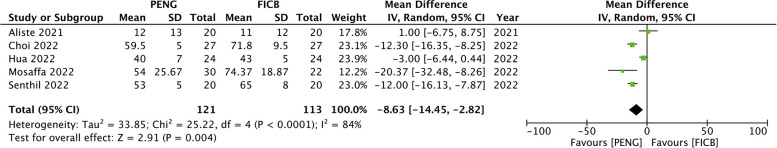
Meta-analysis of 24 h total analgesic consumption between PENG and FICB groups.

Five trials reported data on 24-h total analgesic consumption. Meta-analysis showed that mean opioid consumption in morphine equivalents was significantly less with PENG as compared to FICB (MD: −8.63 95% CI: −14.45, −2.82 *I*^2 ^= 84% *p* = 0.004) (Figure 3). The result was unchanged on sensitivity analysis.

**Figure 4:**
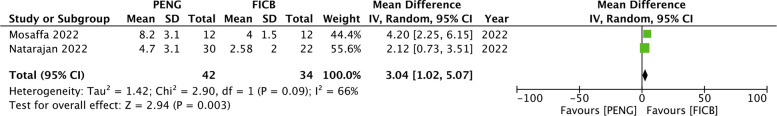
Meta-analysis of time to first analgesic request between PENG and FICB groups.

**Figure 5:**
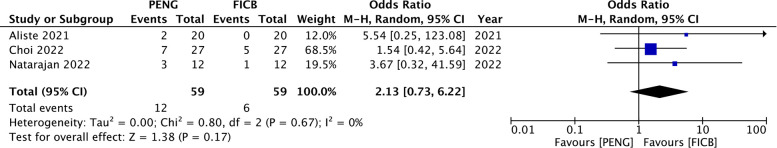
Meta-analysis of PONV between PENG and FICB groups.

Only two studies presented data on time to the first analgesic request. Meta-analysis showed a significantly longer time to first analgesic request in hours for patients given PENG as compared to FICB (MD: 3.04 95% CI: 1.02, 5.07 *I*^2 ^= 66% *p* = 0.003) (Figure 4). Complication data was not uniformly presented by the trials. A meta-analysis of only postoperative nausea and vomiting (PONV) could be conducted. Pooled analysis from three RCTs showed no difference in the risk of PONV between PENG and FICB groups (OR: 2.13 95% CI: 0.73, 6.22 *I*^2 ^= 0% *p* = 0.17) (Figure 5). The result was unchanged on sensitivity analysis.

### Subgroup analysis

Results of subgroup analysis based on type of FICB are shown in Table 2. Sufficient data was available for subgroup analysis of pain at 6 h, 24 h, and total analgesic consumption. Similar to the primary analysis, subgroup analysis also showed no difference in pain scores between either types of FICB and PENG. However, for total analgesic consumption, mean opioid consumption in morphine equivalents was significantly less with PENG as compared to infrainguinal block but not with suprainguinal block.

**Table 2:** Subgroup analysis based on type of fascia iliaca compartment block.

Variable	Group	Studies	Outcome
Pain 6 h	Suprainguinal	2	MD: −0.80 95% CI: −2.70, 1.11 *I*^2 ^= 92%
	Infrainguinal	3	MD: 0.13 95% CI: −0.36, 0.62 *I*^2 ^= 57%
Pain 24 h	Suprainguinal	2	MD: −0.26 95% CI: −2.17, 1.65 *I*^2 ^= 95%
	Infrainguinal	2	MD: 0.45 95% CI: −0.24, 1.14 *I*^2 ^= 81%
Total analgesic consumption	Suprainguinal	2	MD: −6.08 95% CI: −19.08, 6.93 *I*^2 ^= 89%
	Infrainguinal	3	MD: −10.35 95% CI: −18.81, −1.90 *I*^2 ^= 87%

MD, mean difference; CI, confidence intervals.

### Risk of bias

**Figure 6:**
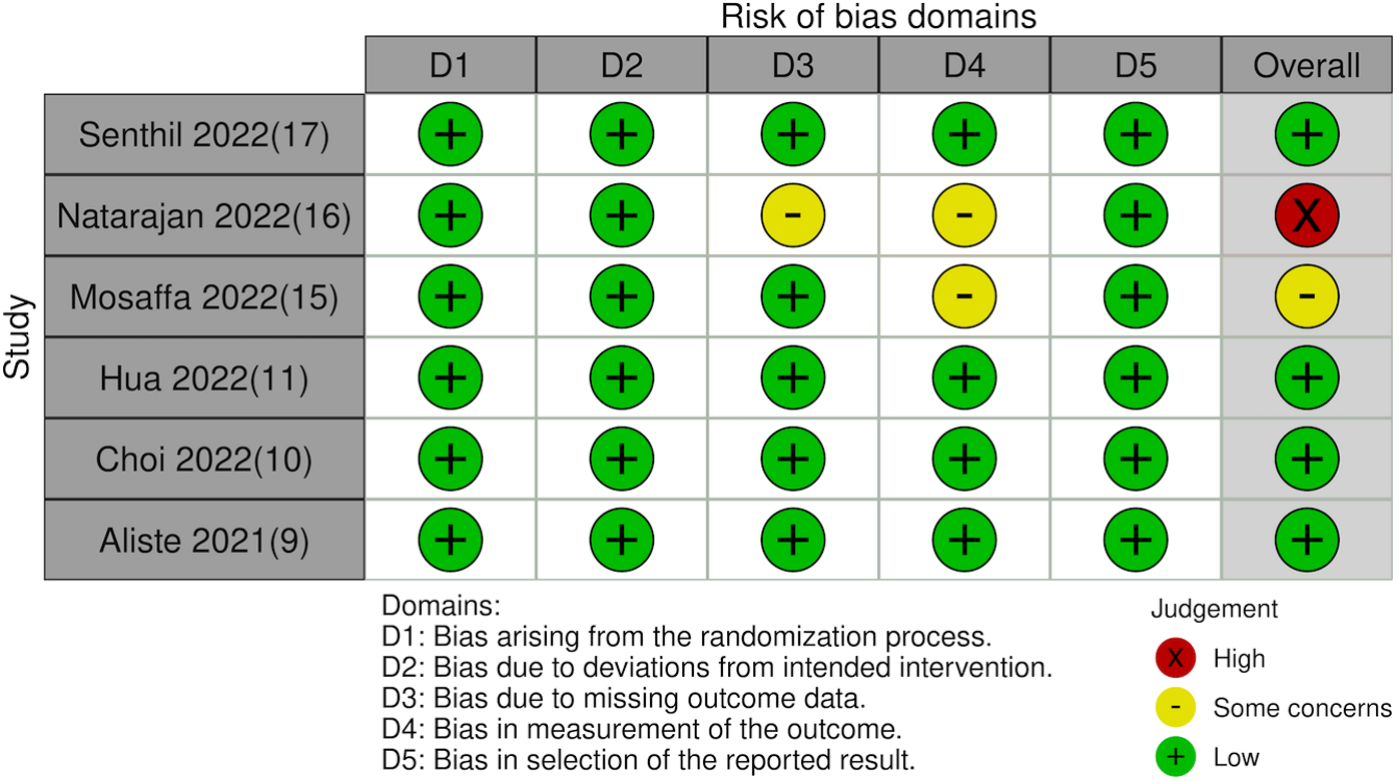
Risk of bias plot.

The risk of bias analysis of the studies is shown in Table 3. Risk of bias plot is presented as Figure 6. All except two trials (16, 17) had a low overall risk of bias.

**Table 3:** Risk of bias in included studies.

Study	Randomization process	Deviation from intended intervention	Missing outcome data	Measurement of outcomes	Selection of reported result	Overall risk of bias
Senthil 2022 (18)	Low risk	Low risk	Low risk	Low risk	Low risk	Low risk
Natarajan 2022 (17)	Low risk	Low risk	Some concerns	Some concerns	Low risk	High risk
Mosaffa 2022 (16)	Low risk	Low risk	Low risk	Some concerns	Low risk	Some concerns
Hua 2022 (11)	Low risk	Low risk	Low risk	Low risk	Low risk	Low risk
Choi 2022 (10)	Low risk	Low risk	Low risk	Low risk	Low risk	Low risk
Aliste 2021 (9)	Low risk	Low risk	Low risk	Low risk	Low risk	Low risk

### Certainty of evidence

Details on the certainty of the evidence is presented in Supplementary Table S2. The certainty of the evidence was moderate for all pain scores and 24 h total analgesic consumption on GRADE assessment. The evidence was downgraded owing to serious concerns for imprecision which was due to high heterogeneity amongst the studies.

The certainty of the evidence was very low for time to first analgesic request. Evidence was downgraded as included trials had concerns regarding blinding of outcome and the analysis had high heterogeneity.

For PONV, the certainty of the evidence was moderate. Evidence was downgraded to due high risk of bias in the trial of Natrajan et al. (17).

## Discussion

To summarize, the meta-analysis showed that PENG block results in significantly reduced total analgesic consumption within 24 h of hip surgeries as compared to FICB. There seems to be no difference in pain scores within 24 h with PENG or FICB. Data on time to first analgesic request and complications was very scarce.

The hip joint has a complex innervation making it a challenge to provide optimal anesthesia post-injury and post-surgery, especially in cases of hip fracture. Research has shown that the anterior capsule is principally supplied by nociceptive fibers while the posterior capsule receives innervation for mechanoreceptors with no sensory fibers (19). The anterior capsule of the hip joint is highly innervated and is the primary source of pain after hip surgeries. Since the nerve supply of the capsule is derived from the femoral nerve, obturator nerve, and accessory obturator nerve, they have been the primary target of regional anesthesia for hip surgeries (20). Traditionally, the FICB has been commonly used for analgesia after different hip surgeries and has been recommended by PROSPECT as the most effective block (21). Nevertheless, there have been questions raised on the efficacy of FICB in blocking the obturator nerve (22, 23). A magnetic resonance imaging study has shown that the injectate distribution to the obturator nerve is limited after FICB (22). Considering these findings, the true anesthetic capability of FICB was questioned with efforts directed to develop a new block that consistently blocked all three nerves (24). The PENG block was developed as an interfascial plane block wherein the local anesthetic is deposited beneath the iliopsoas tendon to target the femoral nerve, obturator nerve, and accessory obturator nerve. Theoretically, the PENG has advantages over traditional regional anesthetic techniques by providing a larger and complete coverage of sensory innervation to the hip which can potentially reduce opioid consumption. Also, the landmarks of the PENG block namely, the anterior inferior iliac spine, the psoas tendon, and the iliopubic eminence are easily identifiable on USG making it technically feasible like other nerve blocks (25).

Considering that there have been no reviews comparing the new PENG block vs. the traditional FICB, our study presents important evidence for clinicians involved with hip surgeries. Our review demonstrated that postoperative pain can be controlled with either the PENG or the FICB with no difference in pain scores within the first 24 h. However, 24-h total analgesic consumption was significantly less with the use of PENG block. Scarce data however failed to indicate any difference in opioid-related adverse events like PONV between the two groups. Furthermore, we were also not able to synthesize much evidence on time for the first analgesic request due to scarce and low-quality evidence. Consistent with our results, a trial by Jadon et al. (26) has shown that PENG block is more effective than FICB for patient positioning for spinal anesthesia in hip fracture patients. Another study by Lin et al. (27) has shown that PENG is superior to femoral nerve block in patients undergoing hip fracture surgeries.

The suprainguinal FICB has been found superior to infrainguinal block in terms of analgesic efficacy (3). To assess the impact of this important variation, a subgroup analysis was performed. It was noted that PENG resulted in significant reduction of total analgesic consumption only in comparison with infrainguinal FICB and there was no difference in analgesic consumption when compared to suprainguinal FICB. While this suggests the superior analgesic efficacy of suprainguinal FICB, the number of studies were too small to draw definitive conclusions and further RCTs are needed.

An important limitation of our review was the inability to compare the motor weakness between the PENG block and FICB. This was primarily due to the unavailability of data from half of the studies and the variability of reporting in the remaining studies. Aliste et al. (9) compared knee extension capability on a 3-point scale between patients receiving the PENG block and FICB. The authors noted significantly better preservation of motor function at 3 h and 6 h with PENG block as compared to FICB. Choi et al. (10) measured quadriceps muscle strength between the two groups using a handheld dynamometer. The muscle strength measured in kilogram-force unit was not significantly different at 6, 24, and 36 h between the PENG and FICB groups. Senthil et al. (18) compared quadriceps power between the two groups using the muscle power scale wherein no quadriceps contraction was taken as 0, for an active anti-gravity movement the grading was 3, and for normal power it was 6. There was no difference between the two groups at 2,6, 10, and 14 h but with slightly better muscle strength at 18 and 24 h. Considering the above findings, it is difficult to conclude that PENG results in compete preservation of motor function during hip surgeries. Indeed, studies indicate that PENG can lead to quadriceps weakness in >25% of cases. This could be due to inadvertent iliopectineal bursal injection during placement of the needle deep to the iliopsoas tendon. Such injection can lead to bursal rupture/puncture followed by an anteromedial spread of the injectate to involve the femoral nerve proper within the fascia iliaca compartment (28). Pascarella et al. (29) have suggested that high volume of local anesthetic even with correct placement of the needle and inadvertent intramuscular injection into the iliopsoas muscle are also possible causes of involvement of the femoral nerve leading to motor weakness with PENG. Indeed, the current evidence is limited and further studies are needed to assess the motor-sparing ability of the PENG block.

Other limitations of our review are as follows. Firstly, only six trials of a limited sample size were available for meta-analysis in the review. Also, the trials did not consistently report all outcomes which reduced the number of studies in each meta-analysis. Secondly, there was high heterogeneity in most of the meta-analyses. This could be due to differences in the study populations, surgical procedures, baseline anesthetic protocols, baseline analgesic drugs, as well as in the type and concentration of local anesthetics used for the blocks. Owing to the small number of studies and much variability amongst the trials we were unable to conduct subgroup analyses for the same. Lastly, most of the trials were from Asian countries with limited data from American or European populations. This prohibits the generalizability of evidence obtained in this review.

The strength of the study is that it is the first systematic review and meta-analysis comparing the PENG block with FICB. Only RCTs were included in our review to generate high-quality evidence. A sensitivity analysis was done to examine the direction of each study on the results. GRADE assessment of the evidence was also carried out to provide certainty of evidence to the readers.

Lastly, it is reiterated that research on PENG is still limited and further avenues still need to be explored. One direction is the utilization of USG-guided posterior pericapsular analgesia in combination with PENG block. Indeed, PENG covers most of the sensory innervation to the hip (25), however, it still lacks complete coverage to the posterior compartment, which draws its innervation from the nerve to the quadratus femoris and superior gluteal nerve (30). Recently, Del Buono et al. (30) have suggested that USG-guided posterior pericapsular analgesia may be used with PENG to provide complete analgesia for hip surgeries. Future RCTs assessing this combination can provide interesting evidence.

## Conclusions

Moderate quality of evidence suggests that PENG may result in better analgesia than FICB in patients undergoing hip surgeries. Data on motor-sparing ability and complications are scarce to draw conclusions. The current review may be considered as preliminary evidence and further large-scale and high-quality RCTs focusing on specific type of surgical interventions and homogenous comparative groups are needed to supplement current findings.

## Data Availability

The original contributions presented in the study are included in the article/**Supplementary Material**, further inquiries can be directed to the corresponding author/s.
