# Supplementary material for: Efficacy of pericapsular nerve group block vs. fascia iliaca compartment block for Hip surgeries: A systematic review and meta-analysis
Source: Front Surg. 2023 Feb 10;10:1054403. doi: 10.3389/fsurg.2023.1054403 (PMC9953592; doi:10.3389/fsurg.2023.1054403)
Supplement: Supplementary file 1 [file Table1.docx]

Supplementary Table 1: Search strategy

| **Query** | **Search Details** |
| --- | --- |
| ((pericapsular nerve group block) AND (fascia iliaca compartment block)) AND (hip) | "pericapsular"[All Fields] AND ("nerve"[All Fields] OR "nerve s"[All Fields] OR "nerved"[All Fields] OR "nerves"[All Fields]) AND ("group s"[All Fields] OR "grouped"[All Fields] OR "grouping"[All Fields] OR "groupings"[All Fields] OR "groups"[All Fields] OR "groups s"[All Fields] OR "population groups"[MeSH Terms] OR ("population"[All Fields] AND "groups"[All Fields]) OR "population groups"[All Fields] OR "group"[All Fields]) AND ("block"[All Fields] OR "blocked"[All Fields] OR "blocking"[All Fields] OR "blockings"[All Fields] OR "blocks"[All Fields]) AND (("fascia"[MeSH Terms] OR "fascia"[All Fields] OR "fasciae"[All Fields] OR "fascias"[All Fields]) AND "iliaca"[All Fields] AND ("compartment"[All Fields] OR "compartment s"[All Fields] OR "compartments"[All Fields]) AND ("block"[All Fields] OR "blocked"[All Fields] OR "blocking"[All Fields] OR "blockings"[All Fields] OR "blocks"[All Fields])) AND ("hip"[MeSH Terms] OR "hip"[All Fields]) |
| (fascia iliaca compartment block) AND (surgery) | ("fascia"[MeSH Terms] OR "fascia"[All Fields] OR "fasciae"[All Fields] OR "fascias"[All Fields]) AND "iliaca"[All Fields] AND ("compartment"[All Fields] OR "compartment s"[All Fields] OR "compartments"[All Fields]) AND ("block"[All Fields] OR "blocked"[All Fields] OR "blocking"[All Fields] OR "blockings"[All Fields] OR "blocks"[All Fields]) AND ("surgery"[MeSH Subheading] OR "surgery"[All Fields] OR "surgical procedures, operative"[MeSH Terms] OR ("surgical"[All Fields] AND "procedures"[All Fields] AND "operative"[All Fields]) OR "operative surgical procedures"[All Fields] OR "general surgery"[MeSH Terms] OR ("general"[All Fields] AND "surgery"[All Fields]) OR "general surgery"[All Fields] OR "surgery s"[All Fields] OR "surgerys"[All Fields] OR "surgeries"[All Fields]) |
| (pericapsular nerve group block) AND (surgery) | "pericapsular"[All Fields] AND ("nerve"[All Fields] OR "nerve s"[All Fields] OR "nerved"[All Fields] OR "nerves"[All Fields]) AND ("group s"[All Fields] OR "grouped"[All Fields] OR "grouping"[All Fields] OR "groupings"[All Fields] OR "groups"[All Fields] OR "groups s"[All Fields] OR "population groups"[MeSH Terms] OR ("population"[All Fields] AND "groups"[All Fields]) OR "population groups"[All Fields] OR "group"[All Fields]) AND ("block"[All Fields] OR "blocked"[All Fields] OR "blocking"[All Fields] OR "blockings"[All Fields] OR "blocks"[All Fields]) AND ("surgery"[MeSH Subheading] OR "surgery"[All Fields] OR "surgical procedures, operative"[MeSH Terms] OR ("surgical"[All Fields] AND "procedures"[All Fields] AND "operative"[All Fields]) OR "operative surgical procedures"[All Fields] OR "general surgery"[MeSH Terms] OR ("general"[All Fields] AND "surgery"[All Fields]) OR "general surgery"[All Fields] OR "surgery s"[All Fields] OR "surgerys"[All Fields] OR "surgeries"[All Fields]) |
| (PENG) AND (hip) | "PENG"[All Fields] AND ("hip"[MeSH Terms] OR "hip"[All Fields]) |
