# Supplementary material for: Efficacy of pericapsular nerve group block vs. fascia iliaca compartment block for Hip surgeries: A systematic review and meta-analysis
Source: Front Surg. 2023 Feb 10;10:1054403. doi: 10.3389/fsurg.2023.1054403 (PMC9953592; doi:10.3389/fsurg.2023.1054403)
Supplement: Supplementary file 2 [file Table2.docx]

| **Supplementary Table 2: GRADE assessment of evidence** | | | | | | | | | | | |
| --- | --- | --- | --- | --- | --- | --- | --- | --- | --- | --- | --- |
| **Certainty assessment** | | | | | | | **Summary of findings** | | | | |
| **Participants (studies) Follow-up** | **Risk of bias** | **Inconsistency** | **Indirectness** | **Imprecision** | **Publication bias** | **Overall certainty of evidence** | **Study event rates (%)** | | **Relative effect (95% CI)** | **Anticipated absolute effects** | |
|  |  |  |  |  |  |  | **With FICB** | **With PENG** |  | **Risk with FICB** | **Risk difference with PENG** |
| **Pain - 6 hours** | | | | | | | | | | | |
| 234 (5 RCTs) | not serious | not serious | not serious | serious^a^ | none | ⨁⨁⨁◯ Moderate | 113 | 121 | - | The mean pain - 6 hours was **0** | MD **0.19 lower** (1.18 lower to 0.79 higher) |
| **Pain - 12 hours** | | | | | | | | | | | |
| 180 (4 RCTs) | not serious | not serious | not serious | serious^a^ | none | ⨁⨁⨁◯ Moderate | 86 | 94 | - | The mean pain - 12 hours was **0** | MD **0.04 higher** (0.44 lower to 0.52 higher) |
| **Pain - 24 hours** | | | | | | | | | | | |
| 182 (4 RCTs) | not serious | not serious | not serious | serious^a^ | none | ⨁⨁⨁◯ Moderate | 91 | 91 | - | The mean pain - 24 hours was **0** | MD **0.09 higher** (1.03 lower to 1.21 higher) |
| **Total analgesic consumption (24 hours)** | | | | | | | | | | | |
| 234 (5 RCTs) | not serious | not serious | not serious | serious^a^ | none | ⨁⨁⨁◯ Moderate | 113 | 121 | - | The mean total analgesic consumption was **0** | MD **8.63 lower** (14.45 lower to 2.82 lower) |
| **Time for first analgesic request** | | | | | | | | | | | |
| 76 (2 RCTs) | very serious^b^ | not serious | not serious | serious^a^ | none | ⨁◯◯◯ Very low | 34 | 42 | - | The mean time for first analg was **0** | MD **3.04 higher** (1.02 higher to 5.07 higher) |
| **PONV** | | | | | | | | | | | |
| 118 (3 RCTs) | serious^c^ | not serious | not serious | not serious | none | ⨁⨁⨁◯ Moderate | 6/59 (10.2%) | 12/59 (20.3%) | **OR 2.13** (0.73 to 6.22) | 102 per 1,000 | **93 more per 1,000** (from 25 fewer to 312 more) |

**CI:** confidence interval; **MD:** mean difference; **OR:** odds ratio

#### Explanations

a. High heterogeneity in the meta-analysis

b. Both included trials had concerns regarding blinding of outcome assessment

c. High risk of bias in Natarajan et al
